# Quantitative and Qualitative Analysis of Agricultural Fields Based on Aerial Multispectral Images Using Neural Networks

**DOI:** 10.3390/s23229251

**Published:** 2023-11-17

**Authors:** Krzysztof Strzępek, Mateusz Salach, Bartosz Trybus, Karol Siwiec, Bartosz Pawłowicz, Andrzej Paszkiewicz

**Affiliations:** 1 The Faculty of Electrical and Computer Engineering, Rzeszow University of Technology, 35-959 Rzeszow, Poland; krzysztof.strzepek@kneiti.prz.edu.pl (K.S.); karol.siwiec@kneiti.prz.edu.pl (K.S.); 2 Department of Complex Systems, Rzeszow University of Technology, 35-959 Rzeszow, Poland; m.salach@prz.edu.pl (M.S.); andrzejp@prz.edu.pl (A.P.); 3 Department of Computer and Control Engineering, Rzeszow University of Technology, 35-959 Rzeszow, Poland; 4 Department of Electronic and Telecommunications Systems, Rzeszow University of Technology, 35-959 Rzeszow, Poland; barpaw@prz.edu.pl

**Keywords:** drones, deep learning, agriculture, neural networks

## Abstract

This article presents an integrated system that uses the capabilities of unmanned aerial vehicles (UAVs) to perform a comprehensive crop analysis, combining qualitative and quantitative evaluations for efficient agricultural management. A convolutional neural network-based model, Detectron2, serves as the foundation for detecting and segmenting objects of interest in acquired aerial images. This model was trained on a dataset prepared using the COCO format, which features a variety of annotated objects. The system architecture comprises a frontend and a backend component. The frontend facilitates user interaction and annotation of objects on multispectral images. The backend involves image loading, project management, polygon handling, and multispectral image processing. For qualitative analysis, users can delineate regions of interest using polygons, which are then subjected to analysis using the Normalized Difference Vegetation Index (NDVI) or Optimized Soil Adjusted Vegetation Index (OSAVI). For quantitative analysis, the system deploys a pre-trained model capable of object detection, allowing for the counting and localization of specific objects, with a focus on young lettuce crops. The prediction quality of the model has been calculated using the AP (Average Precision) metric. The trained neural network exhibited robust performance in detecting objects, even within small images.

## 1. Introduction

Precision agriculture is a concept related to the need for progressive automation and control of cultivation and breeding processes to maximize yields and manage available resources sensibly. With the increase in population, there is also a growing demand for food products, which consequently calls for an increase in food production. In order to enhance the quality and quantity of yields obtained from cultivated fields, the use of unmanned aerial vehicles (UAVs) for conducting plant protection treatments and detailed analysis of cultivated fields using mounted multispectral cameras has become popular [1,2,3].

Multispectral terrain imaging was initiated with the launch of the “Landsat 1” satellite into space by NASA. The satellite was equipped with a multispectral scanner capable of recording four bands of light ranging from 0.5 μm to 1.1 μm [4]. The images captured by the satellite provided a new perspective of the Earth and yielded data that was previously unavailable on a global scale. One significant advantage of these images is their ability to cover large areas with a single photograph; however, a drawback is the limited level of detail in the obtained pictures.

To increase the precision of the images, along with the miniaturization of electronics and the proliferation of unmanned aerial vehicles, multi-rotor flying platforms with mounted multispectral cameras have begun to be used. This enables detailed imaging of soil and plants, providing precise data about the crops. These devices allow for relatively fast and accurate examination of large-scale cultivated fields by capturing multiple images in wavelengths invisible to the human eye.

Through a precise analysis of multispectral images, it is possible to accurately assess the conditions of plants, including determining the vegetation phase, identifying specific pathogens, and evaluating crop quality. Various indicators are used during the assessment of crop quality and vegetation in a particular area. The most popular indicator is NDVI (Normalized Difference Vegetation Index), which is sensitive to the absorption of chlorophyll by plants, thereby allowing the estimation of its content in plants [5].

**Figure 1:**
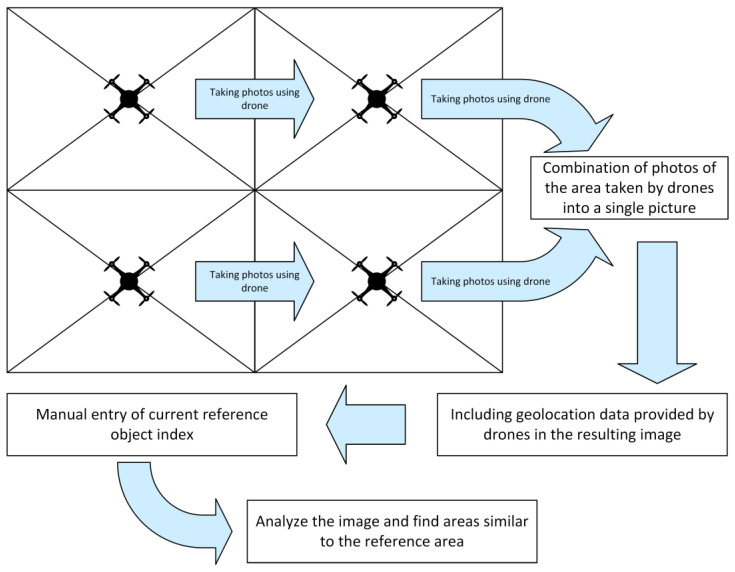
Algorithm for combining images with metadata.

Conducting data analysis on crops using UAVs has become a popular and cost-effective solution among farmers, enabling them to take preventive actions against crop failure. Analyzing the data obtained in this way has a positive impact on the costs of using expensive treatments by allowing targeted spraying only on infected areas rather than treating the entire crop surface. To obtain information useful for farmers, data collected by UAVs must be appropriately processed and subjected to analyses. For this purpose, a specialized procedure is used, aiming to create a large composite image of the entire cultivated field by merging smaller images, each with its individual geolocation data stored in metadata (geotags). On such a prepared image, it is possible to manually input an object index, which serves as the current reference object, and after conducting appropriate calculations, manually identify areas exhibiting pathologies (Figure 1).

The complexity of the entire process and hardware requirements are the reasons for the limited availability of software solutions that enable a satisfactory level of automation in crop research. However, by leveraging the knowledge of deep learning principles and libraries that support multispectral image processing, it is possible to develop software that allows for a partially automated execution of specific analyses. Such software also enables the marking of areas characterized by anomalies and can isolate only specific plant species with minimal user intervention.

Therefore, this paper proposes an innovative approach to the process of monitoring crop quality based on the utilization of georeferenced images analyzed by a properly trained neural network. This approach aims to identify specific objects in the images and estimate the effectiveness of cultivation through the conducted analysis. Section 1 presents the idea of UAVs in agriculture; Section 2 covers similar or different approaches to the presented problem. Section 3 and Section 4 present the implementation of algorithms and usage of Detectron2 while Section 5 focuses on the implementation of the presented idea. Section 6 presents the results and Section 7 summarizes the concept.

## 2. Related Work

Unmanned aerial vehicles (UAVs), commonly known as drones, have diverse applications across various industries. Their adaptability in carrying specialized measurement equipment makes them valuable tools for object recognition and research.

In the realm of research, drones are extensively used for specific tasks, enhancing various domains. Some researchers have devised solutions based on object recognition, employing cameras affixed to UAVs [6,7,8]. Analyzing moving objects within imagery remains an intriguing field of study, with several solutions designed to identify patterns and extract specific data from images for subsequent analysis [9,10]. UAVs are especially vital in scenarios involving peril, such as natural disasters. Various solutions have been developed to aid in locating individuals affected by earthquakes, building collapses, or river floods [11,12,13,14,15,16]. While these solutions use cameras to detect specific objects, they primarily focus on object identification within images, which can vary in size depending on the object being sought. This diversity of objects necessitates complex algorithms, increased computational power for in-depth analysis, and the capacity to identify dissimilar objects.

UAVs also play a significant role in industrial inspections, often leveraging specialized software or hardware [17,18,19]. However, these solutions are tailored to specific tasks and are not versatile for other activities. In the case of multispectral analysis, as proposed here, drones require dedicated multispectral cameras to capture the required images. Multispectral cameras are instrumental in object classification [20,21,22] and pattern analysis [23], and when combined with advanced deep learning algorithms [1,24,25,26,27,28,29], they can provide substantial data for comprehensive analysis when used effectively.

Furthermore, UAVs are indispensable for inspecting agricultural fields [30,31,32,33,34]. Many individuals employ algorithms and models to estimate NDVI (Normalized Difference Vegetation Index) using images from multispectral cameras and aerial platforms equipped with multispectral NDVI imaging systems [35,36]. Some algorithms are designed for route planning or precise mapping through UAVs [37,38]. Other solutions incorporate cameras and sensors to assess land and irrigation practices or to evaluate crop quality [39,40,41,42,43]. While these solutions enable the analysis of crop growth, none of them specifically address the identification of damaged plants. The solution presented in this article centers on identifying unhealthy trees, which are subsequently replaced through reforestation efforts.

## 3. Multispectral Image Processing

Photogrammetric analysis has diverse applications, enabling in-depth examination of acquired images. Satellite images are commonly used to cover large areas, especially in relation to vegetation. This information finds utility in various fields, such as geography, geology, cartography, forestry, and agriculture. In agriculture and forestry, it allows for easy estimation of vegetation coverage and well-being.

Nevertheless, satellite images come with certain limitations. One notable drawback is their reduced level of detail when focused on a specific scanning area, especially when compared to images captured using UAVs. For instance, the multispectral image resolution for the Sentinel-2 satellite is 10 m per pixel, while a drone’s multispectral image offers much higher detail at 6 cm per pixel. This does not eliminate the possibility of using other images from commercial satellites. However, their cost can be prohibitively high when mapping extensive areas. Therefore, advancements in unmanned aerial vehicle (UAV) technology, electronic miniaturization, and the expanded capabilities of these devices enable customized terrain mapping tailored to the precise needs and preferences of landowners. Scanning can be carried out within specified areas with a predefined level of accuracy.

The project presented by the authors involves the utilization of images captured by a drone equipped with a multispectral camera and leveraging artificial intelligence for the detection of areas and elements that require urgent attention, such as dead or diseased trees or crops. However, the multispectral materials collected during the drone flights must be adequately processed and subjected to analysis beforehand. Advanced software serves this purpose, aiming to create a large composite image from multiple smaller photos, each containing its individual geolocation data stored in metadata. The resulting image is not only detailed but also capable of covering extensive cultivated areas, providing an accurate description and representation. On such created images, one must manually input the index of interest and, after performing appropriate calculations, independently identify areas exhibiting pathologies.

**Figure 2:**
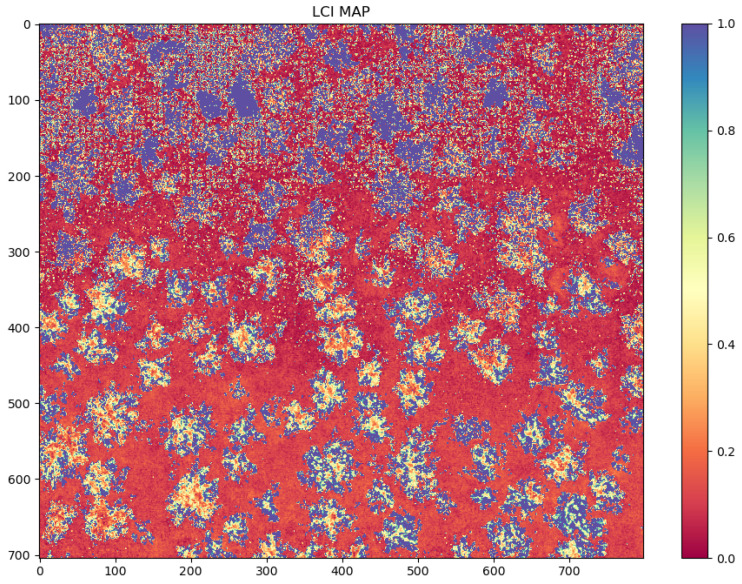
An example of a Leaf Chlorophyll Index (LCI) map.

The currently used systems do not allow for the automatic detection of areas of interest, for example, the detection of a specific pathogen in a given area, but only manual marking with polygons. Such tasks are carried out by experts who analyze maps and manually mark suspected occurrences of plant diseases or create reports on the crop quality status. This process may carry the risk of errors or omitting critical regions due to noise or errors resulting from image registration and atmospheric contamination. Illustrating this situation, Figure 2 shows a generated map of the Leaf Chlorophyll Index (LCI), representing the chlorophyll content in tree leaves. In its lower part, tree canopies with low chlorophyll concentration can be easily isolated, while in the upper part, significant noise can reduce the clarity of the analysis and mislead the analyst.

However, convolutional neural networks can come to the rescue, as they specialize in object detection, segmentation, and classification in images. These techniques can significantly accelerate the process of examining a specific area and can do it more efficiently than an analyst. By leveraging the knowledge of deep learning principles and using available libraries that support multispectral image processing, it is possible to develop software capable of conducting specific analyses, marking areas with anomalies, or isolating specific plant species with minimal user intervention.

The process of transforming raw georeferenced images into a human-interpretable form and subjecting it to analysis is complex. Each image contains metadata marked with specific tags, providing information about coordinates in relation to the adopted georeferencing units, pixel size in relation to the chosen unit of measurement, the number of raster bands with information about reflected light, encoding information, maximum and minimum values, sunlight, cloud cover, and many others. Then, using this metadata, a single high-resolution image covering the entire surveyed area (extent) is created from a set of smaller images for analysis. There can be many more tags, and their quantity and content depend on the camera and the programmer who, during the initial processing and assembly of such an image, can define additional metadata.

Having the single assembled image, one must read the tags to extract information about the layers and their dimensions. Then, individual layer data presented in the form of separate arrays of the original image’s size should be extracted. By doing so, we can generate an image with a specific analysis. Consequently, it becomes possible to create an RGB image or, using appropriate arithmetic operations on individual layers, generate an index map.

In the process of quantitative analysis, the image is fed into a deep neural network, which, thanks to its trained weights, performs object detection on the image, providing information about their coordinates and quantities. As the output of the detection process is a list of polygons with their X and Y coordinates, it is relatively easy to relate them to the actual geolocation coordinates and determine their quantity.

However, to conduct qualitative analyses, appropriate rasters containing information about the registered spectrum of reflected light need to be processed using index-specific formulas, which will yield the desired indexed map.

**Figure 3:**
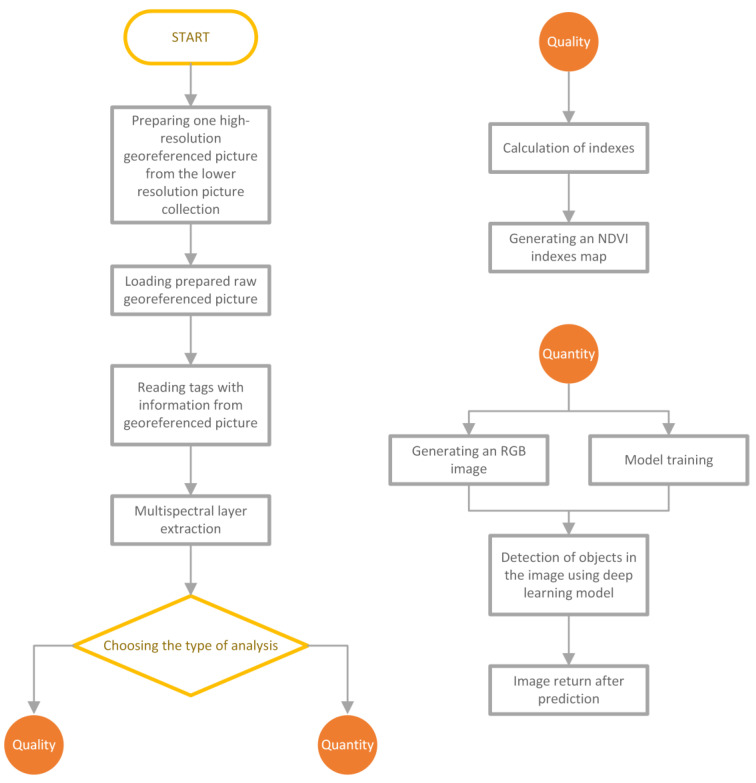
The process of image analysis divided into a qualitative and quantitative model.

The block diagram in Figure 3 lists the steps to be performed in the analysis process.

## 4. Neural Network Training

**Figure 4:**
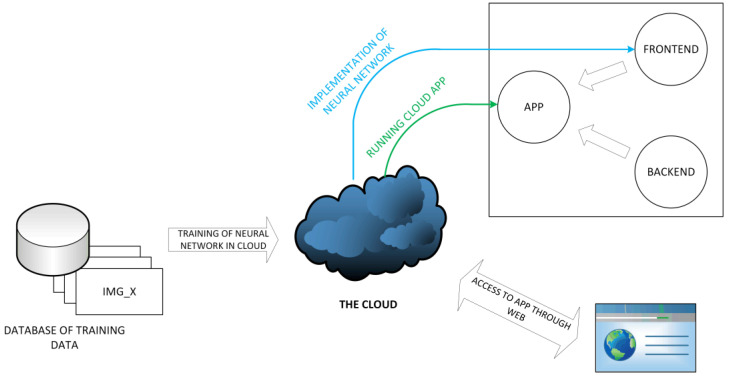
The cloud computing process in the application.

Due to the presence of a neural network in the proposed solution, it was necessary to prepare an appropriate training database and conduct its learning. To efficiently and quickly carry out this process, a powerful computing unit capable of hardware acceleration is required. Cloud solutions offer such a possibility, providing server machines designed for complex computations. Once the neural network’s weights were trained, libraries enabling its implementation and image manipulation were utilized (Figure 4).

**Figure 5:**
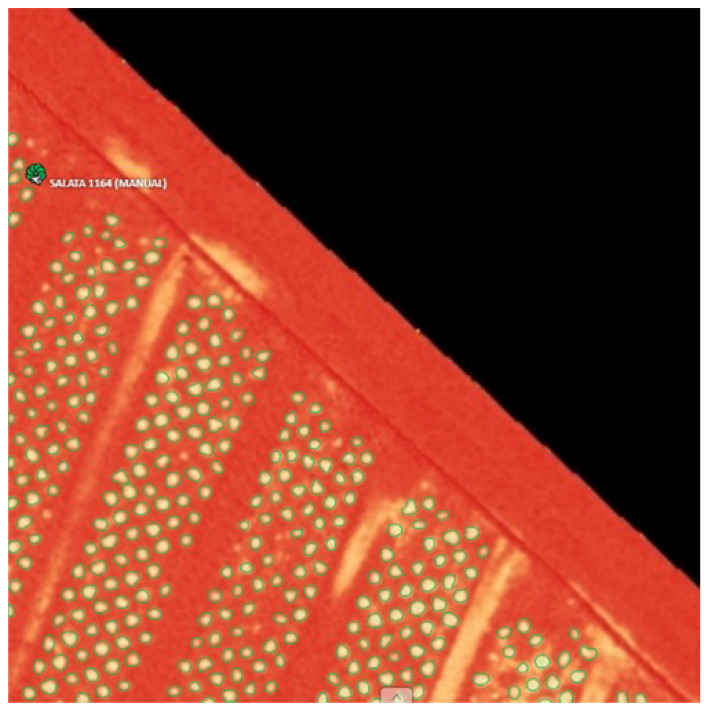
Preparation of the training set in the CVAT software.

For this project, the QT Framework was used for the graphical interface, along with PyCharm and the Python programming language for image analysis, processing, and application logic. The Computer Vision Annotation Tool v1.7.0 (CVAT, Figure 5) was utilized for annotating and classifying objects on images for computer vision and artificial intelligence purposes. The prepared images were then used to develop a training database using data acquired from photogrammetric surveys.

The images were divided into two sets, the training set and the validation set, due to the characteristics of the training process of convolutional neural networks. The training set is used to train the detector so that it activates in the locations where the detected object is present. However, to check if the detector responds correctly, it needs to be tested on a set it has not seen before, namely the validation set. As a result, the training process of the detector can be appropriately manipulated to improve its performance and achieve its task more effectively.

Depending on the type of detection being conducted, several popular formats are used for preparing the training dataset. The Common Objects in Context (COCO) format is the most advanced one. It supports creating bounding boxes, polygons, skeletons, points, and many others, for instance, segmentation, object detection, or skeleton tracking [44]. The Pascal Visual Object Classes (VOC) format is also used, allowing annotations for objects and instance detection like COCO, but the information is stored in an XML file instead of a JSON file [45]. Additionally, there are formats like YOLO and ImageNet. The YOLO format is specifically designed for annotating images for the You Only Look Once network architecture, which is used exclusively for object detection, supporting only bounding box creation. On the other hand, ImageNet’s format is similar to Pascal VOC in usage since annotations are stored in XML, but it supports only object detection. To perform instance segmentation of objects, the prepared training dataset was based on the COCO format.

The learning process was based on the Detectron2 model, an updated version originally based on the Faster R-CNN model, created by the Facebook AI Research team [46].

Detectron2 naturally derives from the Faster R-CNN architecture, whose base detector is built upon the Feature Pyramid Network (FPN) [47]. By utilizing the FPN, Faster R-CNN becomes a multi-scale detector, enabling the detection of objects both small and large. Consequently, it serves as the backbone of the Detectron architecture. Its primary task is to extract feature maps for the input image at various scales. The scales divide the image into 1/4 (P2), 1/8 (P3), 1/16 (P4), 1/32 (P5), and 1/64 (P6) of the original resolution. From each scale layer, the Region Proposal Network (RPN) identifies object regions in the feature map, providing a confidence level for each detection. Next, the Box Head trims the objects and extracts the essence from the feature maps to dimensions proposed by the RPN, forming multi-dimensional features of constant size. Fully connected layers fine-tune the location of bounding boxes and classify the detected objects. Finally, the result of all these operations is 100 (by default) boxes which are filtered using non-maximum suppression [48].

Knowing that the first part of the Detectron2 architecture relies on feature extraction, by downsizing the input image’s scale by subsequent powers of two, images with dimensions of 256 × 256 pixels resulting from cropping the indexed map after the Optimized Soil Adjusted Vegetation Index (OSAVI) analysis were prepared. In this way, a training and validation dataset of five images each was obtained. Despite having a very small training dataset, the annotation process yielded over 1200 instances of objects, and for the validation set, over 800 instances. For research purposes, young butterhead lettuce was selected as the crop for detection.

**Figure 6:**
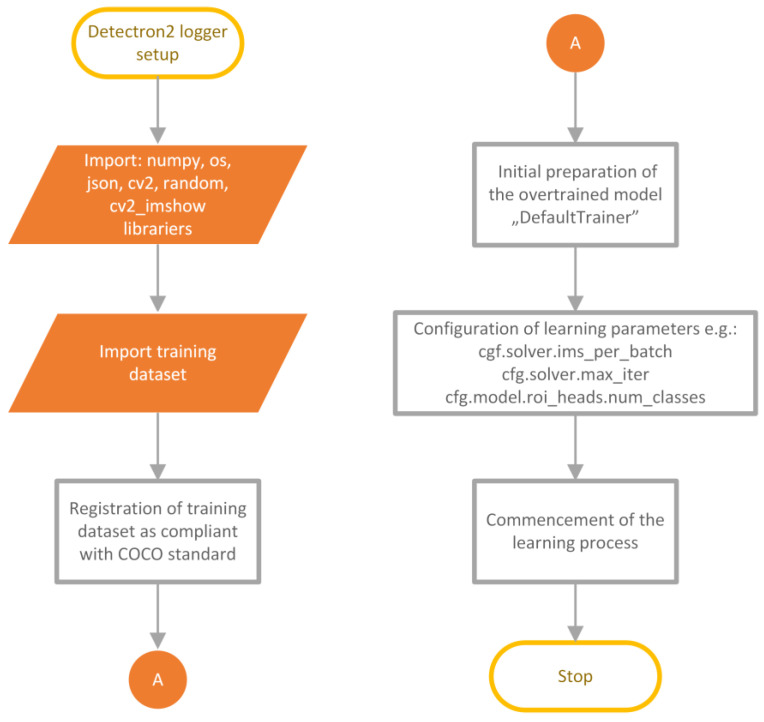
Diagram showing the operation of the Google Colab script for network training.

The utilization of advanced learning and image processing algorithms required powerful computing units. Leveraging hardware acceleration, the learning process took approximately 4h on highly efficient NVIDIA Tesla P4 graphic cards. This process was conducted on the Google Colab platform, which provides an environment and hardware architecture for such tasks. The preparation of the training environment is depicted in Figure 6. It shows the sequential import of the training dataset, its registration as a dataset compatible with the COCO standard [49], the preparation of a pre-trained model provided by the creators of the architecture, the configuration of learning parameters, and the initiation of the entire process. To speed up the learning process, the authors used a method called transfer learning. It involves utilizing a pre-trained model with learned weights and activation tensor layers for certain characteristic features of the image and adapting it to the current training data. This way, the improved model gains additional information that could not be extracted from a small training dataset while also shortening the learning time.

**Figure 7:**
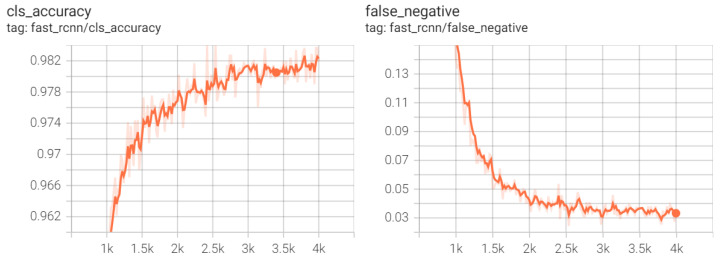
Plots showing the accuracy of the classification of detected objects during training (**left**) and the percentage of false negative samples (**right**).

After the completion of the learning process, the trained weights were saved, and model evaluation was performed. Consequently, plots were generated to visualize how well the network underwent the learning process (Figure 7). The first plot displays the accuracy achieved by the network, which is the ratio of correctly detected objects to the total number of annotated objects, expressed as a percentage. The second metric shown is the percentage of false negatives, which represents objects incorrectly classified as background and belonging to the described class. As observed, the learning process was chaotic, but with each iteration through the network, its parameters improved. This was due to the small number of batch images, resulting in a more stochastic process of improving prediction accuracy. Nonetheless, notable benefits arose from the implementation of transfer learning. After just a thousand iterations, the network achieved an accuracy exceeding 90%, and with each subsequent iteration, its precision continued to increase.

## 5. Implementation

**Figure 8:**
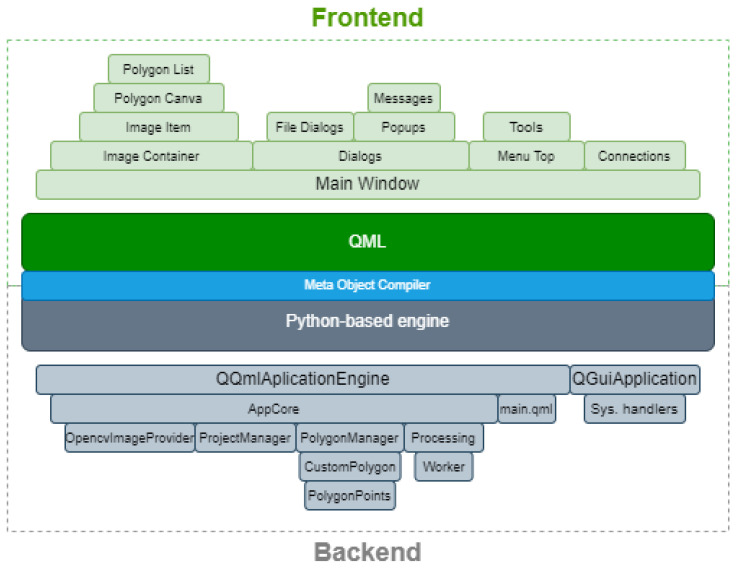
Application classes divided into frontend and backend layers.

Based on the promising learning results, the authors created an application that would facilitate the creation of index maps and conduct quantitative crop analysis. The application consists of a backend and a frontend layer. The backend, written in Python, manages all the logic and analysis processes, implements the functionality of the Detectron2 network architecture, and handles the import and arithmetic operations on multispectral images. The frontend layer is responsible for the user interface and is written in QML, a modification of JavaScript. The Signal-Slot system enables the calling of functions and data transfer between the frontend and backend layers. The architecture of the prepared application is depicted in Figure 8. The logic and interface layers are shown in the diagram, with corresponding classes inheriting from each other.

The frontend layer is based on the main window class, which contains all graphical and interactive elements. There are several key elements defining the operation of the graphical interface and interactions with the user, including project creation and communication with the backend.

- Image Container: Stores an object containing the loaded image and allows interaction with it through mouse cursor movement and scaling. It aggregates the Image Element.
- Image Element: A crucial element responsible for storing, displaying, and creating maps on the graphical interface. It reloads the image and displays it after analysis when the appropriate signal is received from the backend.
- Polygon Canvas: Enables drawing polygons on the image.
- Polygon List: Stores a list of drawn polygons, allowing the user to select areas of the map for analysis.
- Dialogs: Manages dialog windows, pop-ups, and feedback messages. It handles project creation, selection of feedback information files related to the analysis, and other summary windows.
- Menu Top: Contains tools that users can use during interaction with the application.
- Connections: Responsible for managing signals and connections between the frontend and backend as well as signals within classes and elements of the user interface.

The backend layer mainly consists of two key objects. The first one is QGuiApplication, which creates support for system interruptions needed for the application’s functioning. The second one is QQmlApplicationEngine, which launches the application and allows interaction between the frontend and backend. Within it, there is a class called AppCore which aggregates all the other classes operating within the program. It implements the singleton design pattern, which means there is exactly one instance of each class within this main object. In this case, these classes are:

- OpencvImageProvider: Responsible for loading images, storing them in the engine’s memory, and appropriately managing all the results of the conducted analyses. Additionally, it facilitates sending images to the user interface.
- ProjectManager: Used in the initial phase to create a project and later to save it and generate reports from the conducted analyses.
- PolygonManager: Stores data related to specific analyses. For qualitative analysis, it provides the averaged index value for the area, while for quantitative analysis, it provides the number of detected object instances. Its data are used by ProjectManager to generate the appropriate report. It also contains information about the list of polygons and points, which is used to visualize them on the user interface.
- Processing: Implements the entire process of both qualitative and quantitative analyses, including generating index maps and using artificial intelligence for quantitative analysis.

**Figure 9:**
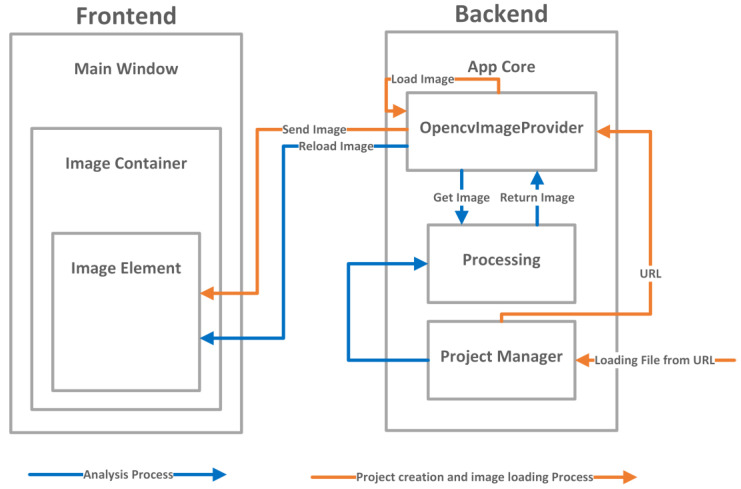
Inter-process communication within the application.

The application uses multiprocessing to create separate processes to optimize the program’s performance and avoid blocking other threads, such as the one responsible for the graphical interface. The architecture of the application with inter-process communication is presented in Figure 9.

**Figure 10:**
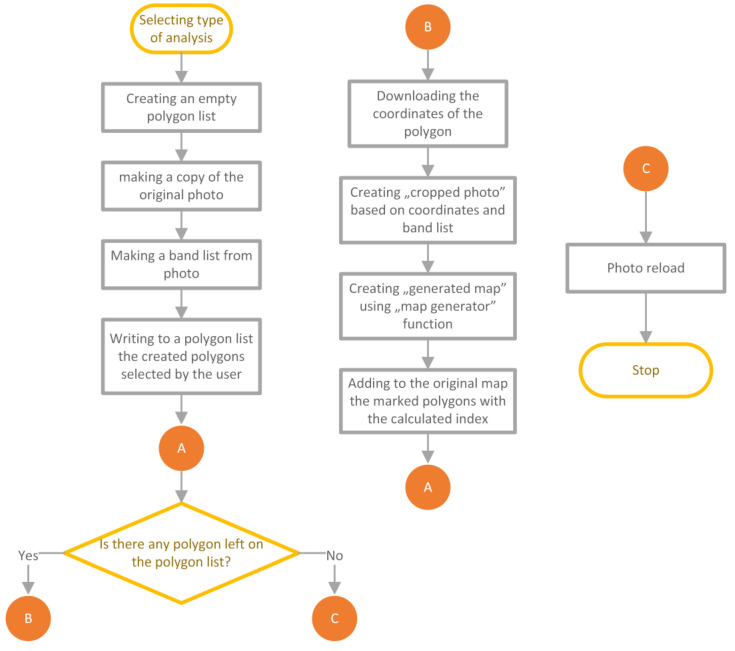
An algorithm creating an index map for a given analysis.

The creation of an index map is done by importing a multispectral map in the .tiff format and processing it within the application engine. For this purpose, the GDAL library is utilized. It supports reading and writing of geospatial raster images, handling various common extensions, and encoding and decoding metadata tags associated with the images. The map is loaded into a list with dimensions W × H × N, where: W (width)—the size of the image horizontally, H (height)—the size of the image vertically, N—the number of registered bands. Depending on the camera used to capture the images, the raster data of a single pixel may be stored as a variable of different sizes, but it is commonly assumed to be stored as int16 type. This prepared list can serve as the source data for further analysis. Then, the red, green, and blue color bands are extracted and combined to form an RGB image, which is more interpretable than a mixed or monochromatic image. By using the OpenCV and Numpy libraries, combining multiple layers into a single image can be achieved quickly through matrix operations. Consequently, a clear terrain map is obtained. Once this preparation process is complete, a corresponding signal is sent to the frontend of the application to reload/load the graphic. This way, an RGB image is generated on the user interface from the previously loaded layers. Utilizing the aforementioned ability to analyze data areas, the user marks the desired area with points to create an appropriate polygon, with its vertices corresponding to specific points on the image and georeferenced on the ground. Upon user confirmation of the requested operation, a specific signal is sent and intercepted by the appropriate slot (which is essentially a function with the corresponding decorator), initiating the background analysis process. By utilizing the multiprocessing library, a separate non-blocking process is created, ensuring that the main interface loop is not affected. The “worker” of this process is provided with the loaded multidimensional list of multispectral images and a list of polygons with areas for analysis. The algorithm of the implemented solution is presented in Figure 10.

After the user indicates the type of analysis, an empty polygon list is created. Then, a copy of the original image is made, and the appropriate bands are retrieved and stored as a list. Next, the user-defined polygons to be analyzed are added to a variable if there are multiple polygons. Then, for each polygon, their coordinates are extracted, and the corresponding photogrammetric bands are extracted from the list and passed to the map_generator() function. For each analysis, this function is customized to the specific index we want to obtain. In this function, an equation is implemented that calculates the index value (e.g., NDVI) for the given pixels using the following formula [5]:

NDVI = (NIR − VIS)/(NIR + VIS)

As a result, a set of pixels with calculated index values is obtained, which are then overlaid on the original image. The final step of the process is emitting signals to reload the image on the frontend layer.

In the case of quantitative analysis, the process is the same up to the point of cropping the bands to the required input size for the detector. In the next step, the appropriate weights are loaded. If these weights are not available in the project folder at the specified path, the user must specify which model to load. With the prepared input data, the model loads the configuration regarding the threshold values at which classification and separation of the detected object from the background are performed, resulting in a list of coordinates with polygons.

Due to the large number of objects in the image, image processing can be very time-consuming as well as consume a large amount of RAM, as observed during testing by the authors of this project. With the mentioned list of coordinates, it is possible to overlay the polygons and their confidence labels on the image for visualization. Additionally, by combining the previously conducted quantitative analysis on a specific area, it is possible to link it to the detected object, thus providing an analysis for each individual plant and determining its health status.

## 6. Results

**Figure 11:**
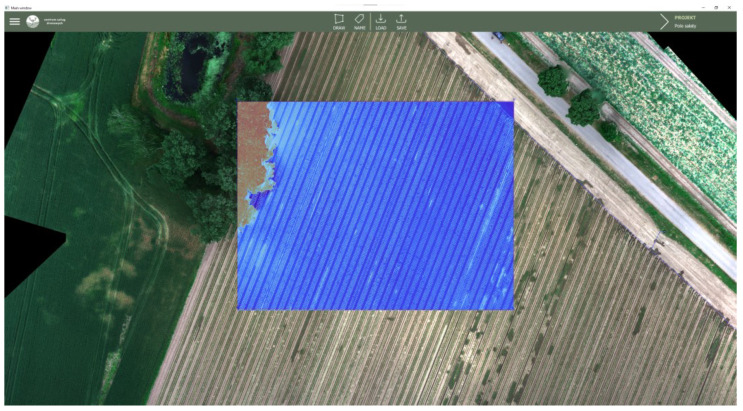
NDVI vegetation index map generated by the application.

The operation of the application for a sample area and the NDVI vegetation index can be observed in Figure 11. As a result of its function, it was possible to load a large georeferenced image file and generate a vegetation index map within the marked square polygon area. At the same time, the application maintained smooth performance, and its operation did not consume significant resources except for the RAM, where the loaded image is stored.

**Figure 12:**
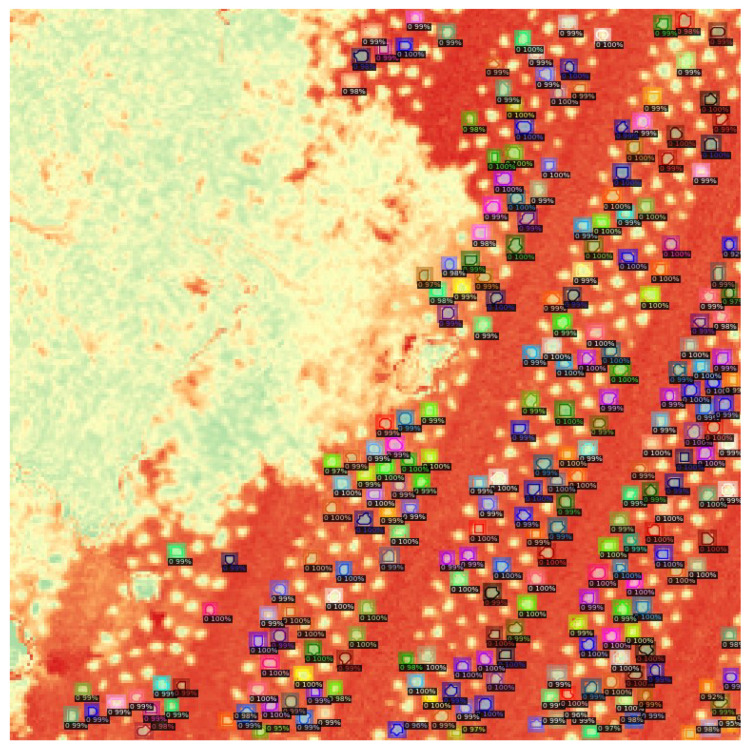
Lettuce detection results on OSAVI map.

In Figure 12, in a large zoom-in view, one can observe how Detectron2 performs object detection and segmentation oriented towards the OSAVI index. Despite the small size of the input image (256 × 256) and the small objects, it was able to correctly recognize and isolate most of them from the background.

In order to assess the quality of the trained model, the AP (Average Precision) metric was employed. This metric is applicable in tasks related to object detection, segmentation, and separation in artificial intelligence models. It not only informs us about whether an object was correctly classified but also measures how close the predicted bounding box and segmentation mask are to the real object’s position, separating it from the background or objects of other classes. In the model evaluation process, it is first tested on a dataset, and then precision and recall are calculated for various decision thresholds. Precision is the ratio of true positive detections (correctly classified) to the sum of true positive and false positive detections. Recall is the ratio of true positive detections to the sum of true positive detections and false negative detections. For each point on the precision–recall curve, interpolation is performed to obtain a smooth curve by replacing precision at a given point with the maximum precision value for any higher recall. AP is calculated as the average precision value after interpolation for all recall values.

Mathematically, AP is computed as the sum of recall differences multiplied by precision at each point:

AP = sum(Rn − Rn^−1^) × Pn

where Rn and Pn represent recall and precision at the n-th point on the curve, and the sum is calculated over all points on the curve.

The AP metrics are calculated for various Intersection over Union (IoU) thresholds. IoU, in turn, represents the ratio of the overlapping area between two segmentation masks or bounding boxes to the sum of these two areas. In other words, if the location prediction is perfect, IoU is equal to 1, and if the prediction does not overlap with the reference at all, IoU is 0. AP50 uses an IoU threshold of 0.5, AP75 uses an IoU threshold of 0.75, and AP95 calculates the average AP across IoU thresholds from 0.5 to 0.95 with a step size of 0.05.

The quality analysis of the trained model consisted of two stages. First, the quality of object bounding box predictions was examined, followed by the assessment of segmentation mask predictions. The results are presented in Table 1. The “AP” column represents the average of all precisions at IoU thresholds from 50 to 95. The next columns show the average accuracy for IoU thresholds of 50% and 75%, and the last column is “AP50:95” for a small detection area.

As observed, the prediction of bounding boxes for IoU 50% and 75% is very good, with accuracies of 89% and 66%, respectively. On the other hand, the prediction of segmentation masks for IoU 50% has a high accuracy of 89%, while for IoU 75%, it is lower at 58%. The reason for this discrepancy may be the size of the detected lettuce in the cultivated area, which averaged around 28 pixels, depending on its growth stage. Therefore, even slight inaccuracies in mask predictions resulted in a significant reduction in the AP metric’s quality.

## 7. Conclusions

The authors of the work have created a system for simultaneous analysis of the quality and quantity of crops. The detector exhibits high confidence in its predictions, but it also shows some shortcomings in generalizing the features of the remaining undetected lettuce. This phenomenon may be attributed to two reasons. First, the dataset used for training might have been too small and should be enlarged and diversified more randomly. Another reason could be overfitting, a situation where the model becomes too specific to the training data, losing its ability to effectively generalize features.

The potential for further development of the application is quite significant. The trained neural network worked on the lettuce dataset, but it can be quickly and efficiently retrained to detect other objects, such as apple tree crowns, beet crops, etc. Furthermore, the network can be fine-tuned to detect specific shapes or arrangements of objects, which can positively influence the quality of the subsequent analysis process. Each individually detected object can undergo individual analysis to identify specific crop pathogens, or this process can be automated to only return diseased plants or crop parts that require specific care. In this way, farmers can have better control over their crops, taking preventive measures against diseases in a timely manner or determining the ideal time for harvesting.

## Figures and Tables

**Table 1:** Prediction quality for bounding boxes and segmentation masks using various AP metrics.

Prediction	AP	AP50	AP75	AP50:95
Bounding boxes	56.096	89.069	66.683	56.096
Segmentation masks	54.253	89.069	58.230	54.253

## Data Availability

Data are contained within the article.
